# Case Report: Functional investigation of the γENaC G532S mutation presenting as mild PHA-1B3

**DOI:** 10.3389/fmed.2025.1605057

**Published:** 2025-09-03

**Authors:** Eleonora Centonze, Meenakshi Girish, Miguel Xavier van Bemmelen, Olivier Staub, Girish Subramaniam, Stephan Kellenberger

**Affiliations:** ^1^Department of Biomedical Sciences, University of Lausanne, Lausanne, Switzerland; ^2^Department of Pediatrics, All India Institute of Medical Sciences, Nagpur, India; ^3^Colors Children Hospital, Nagpur, India

**Keywords:** aldosterone resistance, dehydration, ENaC, SCNN1G, hyperkalemia, hyponatremia, pseudohypoaldosteronism, case report

## Abstract

Pseudohypoaldosteronism type 1 (PHA-1) is a rare genetic disease caused by aldosterone resistance, characterized by severe sodium loss, hyperkalemia, dehydration, and vomiting. The Epithelial Na^+^ Channel (ENaC) is a cation channel that constitutes the rate-limiting step of transepithelial Na^+^ transport in many tissues and regulates blood volume and pressure. Mutations in any of its subunits (α, β, or γ) have been shown to cause PHA-1B. The present investigation is a case study of a 4-month-old female born to consanguineous parents with symptoms suggestive of a form of PHA-1. The child presented with failure to thrive, accompanied by mild hyponatremia and hyperkalemia, together with a normal anion gap metabolic acidosis. Whole exome sequencing, conducted to identify genetic variants, revealed a variant of uncertain significance, the homozygous missense mutation c.1594G > A, p. Gly532Ser in the *SCNN1G* gene, associated with PHA-1B3. To investigate the functional impact of this mutation, *in vitro* electrophysiological and biochemical studies were performed with wild type αβγ and mutant αβγG532S-ENaC. This analysis showed that the γG532S mutation reduced, but did not suppress ENaC expression and activity. The functional observation explains the mild phenotype of this novel *SCNN1G* mutation, which contrasts with the typically severe presentation of autosomal recessive PHA-1B. In our case, the patient showed a positive clinical response to sodium chloride supplementation alone. These findings suggest that certain missense mutations in *SCNN1G* may result in a milder disease course, underscoring the importance of functional studies in understanding genotype–phenotype correlations in PHA-1.

## 1 Introduction

Aldosterone is the main regulator of extracellular volume and salt balance (1). Aldosterone induces Na^+^ absorption and K^+^ excretion by the renal tubule by increasing the activity of several proteins involved in Na^+^ transport. Pseudohypoaldosteronism (PHA) is a condition in which the clinical symptoms suggest an aldosterone deficiency, but the plasma aldosterone levels are normal or elevated, indicating a resistance to actions of aldosterone (2, 3). PHA-1 is a rare genetic disorder usually presenting in the first days after birth with sodium wasting, hyperkalemia, vomiting and severe dehydration (4–6). Many patients also present with respiratory symptoms and growth retardation, and dermatitis is observed in ~30% of the patients (4). PHA-1 exists in two distinct forms. PHA 1A (MIM#177735) is an autosomal dominant, renal disease caused by mineralocorticoid receptor gene defects. It usually causes mild disease that improves with age. The other form, systemic, autosomal recessive PHA-1B (MIM#264350), is caused by mutations of Epithelial Sodium Channel (ENaC) subunits. It is considered to cause more severe and permanent clinical manifestations. ENaC belongs to the ENaC/degenerin family of amiloride-sensitive cation channels. It is expressed in the apical membrane of cells in the kidney’s distal nephron, in the distal colon, lungs, and ducts of exocrine glands, where it mediates Na^+^ absorption and thereby influences extracellular fluid volume and blood pressure (7, 8). ENaC consists of three subunits, α (SCNN1A), β (SCNN1B), and γ (SCNN1G) in a 1:1:1 stoichiometry (9, 10). PHA-1B is sub-divided according to the ENaC subunit in which the mutation occurs, into PHA-1B1, PHA-2, and PHA-3 (Online Mendelian Inheritance in Man, OMIM^®^. McKusick-Nathans Institute of Genetic Medicine, Johns Hopkins University, Baltimore, MD).^1^ ENaC subunits share a similar structure, featuring cytoplasmic N- and C-termini, an extracellular loop and two transmembrane domains (9, 10), all essential for proper channel function. In the distal nephron, aldosterone controls the sodium reabsorption activity of ENaC by triggering αENaC gene transcription and by redistributing ENaC subunits from intracellular pools to the apical membrane of principal cells (11–13). Mutations causing PHA-1B, leading to renal salt loss and high sodium leakage in sweat, feces, and saliva, have been found in α-, β- and γENaC subunits (14, 15). For some of these mutations it was shown that they decrease or abolish ENaC function (14, 16). PHA-1B3 is caused by homozygous mutations in the γENaC subunit, which disrupt sodium reabsorption and contribute to severe clinical manifestations of the disorder (17). While a mild form has been described in the case of a missense mutation in αENaC (PHA-1B1) (18), a mild phenotype of PHA-1B due to a mutation in β or γ subunits has not been observed so far. We report what we believe to be the first such case, a mild case of PHA-1B due to a missense mutation in the γENaC gene confirmed by functional analysis.

## 2 Case description

A 4-month-old female presented with failure to gain weight. The infant was the second child of a third-degree consanguineous marriage, delivered at full term, with a birth weight of 2.5 kg, and was exclusively breastfed until 5 weeks of age, at which point the infant was transitioned to mixed feeding due to inadequate weight gain. The infant did not appear sick or dehydrated, was normotensive and did not have skin hyperpigmentation. The initial laboratory findings showed hyponatremia (116.3 meq/L) with increased urinary sodium (55.60 meq/L) and mild hyperkalemia (5.47 meq/L). Further investigations for adrenal insufficiency revealed a normal 17-hydroxyprogesterone (17OHP; 5.797 ng/mL), increased cortisol (8 am value 601.61 ng/mL; normal 54.94–287.56), and normal ACTH (15 pg/mL; normal 7.2–63.6). Blood gas analysis revealed a normal anion gap metabolic acidosis. Together with the hyperkalemia, this finding suggested type 4 renal tubular acidosis, which is associated with either aldosterone deficiency or aldosterone resistance. Aldosterone and plasma renin activity (PRA) levels were found to be markedly elevated (aldosterone: 307 ng/dL, normal range 5–90; PRA: 90.65 ng/mL/h; normal range 2.4–37 ng/mL/h), suggesting PHA-1. Given the history of consanguinity, whole exome sequencing was performed, revealing a homozygous missense mutation in the *SCNN1G* gene, with the variant c.1594G > A, p. Gly532Ser, consistent with the PHA-1B3 subtype; this variant is classified as a variant of unknown significance. In gnomAD, the allele frequency of this variant is indicated as 2.5·10^**−6**^. PHA-1B3 is typically a severe disease with life-threatening hyperkalemia in the neonatal period. Our patient had mild hyperkalemia and, unlike typical PHA-1B3, had already shown improvement with just salt supplementation at a dose of 10 meq/kg daily. A similar mild form of autosomal recessive PHA-1B1 has been described with novel missense mutations in the *SCNN1A* gene. As the clinical phenotype was mild and not consistent with the uniformly reported severe manifestations of PHA 1B, *in vitro* functional analysis was performed to understand the discrepancy in the phenotype and the genotype reported.

## 3 Materials and methods

### 3.1 Mutagenesis and RNA synthesis

The experiments were carried out with human ENaC. α-ENaC was transcribed from the pSDEasy vector, which was linearized with BglII (Cat #R0144L, New England Biolabs). β-ENaC was transcribed from the pBSK(+) Xglob construct, and γENaC from the pSD5 vector, both of which were linearized with XbaI (Cat #R0145S, NEB). The G532S mutation in γENaC was introduced using the QuikChange site-directed mutagenesis approach, utilizing the KAPA HiFi HotStart PCR polymerase (Cat #KK2501, KAPA Biosystems). Verification of the mutation’s presence was conducted by sequencing (Microsynth). Capped RNA was synthesized *in vitro* using the mMessage mMachine SP6 or T7 kit (Invitrogen™, Cat #AM1340 and #AM1344). Transcription of α-ENaC and γENaC was done with the SP6 RNA polymerase, while the T7 RNA polymerase was used for β-ENaC.

### 3.2 Electrophysiology and analysis

All procedures with *Xenopus laevis* frogs were approved by the Veterinarian Office of the Canton of Vaud. 1.3 g/L MS-222 (Cat #A5040-250G, Sigma) was used to anaesthetize female *Xenopus laevis* frogs. The oocytes were obtained via a small incision (approximately 1 cm) made in the lateral abdominal region. Subsequently, the ovary lobes were incubated with a collagenase solution (Cat #C9891, Sigma-Aldrich), which contained 1 mg/mL of the enzyme, diluted in a calcium-free modified Barth’s (MBS) solution. This treatment was conducted for a duration of 90 min at room temperature, with the aim of isolating and defolliculating oocytes of stage V and stage VI. Equal concentrations of cRNAs for α-, β- and γENaC subunits were mixed, resulting in a final concentration of 15 ng/μl, with 40 nL cRNA solution injected per oocyte. The oocytes were maintained at 19 °C in MBS solution containing (in mM): 85 NaCl, 1 KCl, 2.4 NaHCO_3_, 0.33 Ca(NO_3_)_2_, 0.82 MgSO_4_, 0.41 CaCl_2_, 10 HEPES, and 4.08 NaOH. Electrophysiological measurements were conducted 20–30 h after the cRNA injection in the oocytes. Currents were recorded using the two-electrode voltage-clamp technique at a holding potential of −80 mV. A Dagan TEV200 amplifier (Minneapolis, MN) equipped with two bath electrodes was used for recordings, operated via PatchMaster (RRID: SCR_000034, HEKA Elektronik-Harvard Bioscience), and analyzed using FitMaster (RRID: SCR_016233, HEKA Elektronik-Harvard Bioscience). Each oocyte was placed in the recording chamber, penetrated by two glass electrodes, each having a resistance below 1 MΩ when filled with 1 M KCl. The recording solutions comprised (in mM): 120 NaCl, 2.5 KCl, 10 HEPES, and 1.8 CaCl_2_. Variants consisted of using this solution either as it was or supplemented with 10 μM amiloride (Cat #A7410, Sigma-Aldrich) or 5 μg/mL trypsin (Cat #T1426, Sigma-Aldrich). The pH was adjusted to 7.4 with NaOH. Current sensitive to amiloride was established through the subtraction of the current measured when 10 μM amiloride was applied from the current measured in its absence. Oocytes expressing WT and γG532S-ENaC were recorded alternately to prevent bias resulting from the increase in ENaC current during the expression period.

### 3.3 Cell-surface biotinylation of oocytes

Oocyte biotinylation and isolation of biotinylated fractions were performed as described previously (19). Control non-injected, or injected oocytes (~20 per condition) were incubated for 15 min on ice in 1 mL Biotinylation buffer (in mM, triethanolamine 10, NaCl 150 mM, CaCl_2_ 2, pH 9.5, supplemented with 1 mg/mL NHS-Sulfo-S-S-Biotin (Thermo-Scientific #21331)). The residual reagent was quenched by replacing the biotinylation solution with 1 mL of MBS supplemented with (in mM) glycine 192, Tris/HCl 25, pH 7.5, and 5 min incubation at 22 °C. After one rinsing step in MBS, the drained oocytes were stored at −20 °C or used immediately.

### 3.4 Isolation of membrane-enriched and surface-biotinylated fractions

To isolate membrane fractions, the oocytes were disrupted by pipetting in 0.75 mL of membrane isolation buffer (in mM): 50 Tris/HCl (pH 7.0 at room temperature), 150 NaCl, 5 MgCl_2_, 10 N-ethylmaleimide, supplemented with cOmplete protease inhibitor cocktail (ULTRA Tablets, Mini, EDTA-free, Roche#05–892–791-001, one tablet per 10 mL), followed by centrifugation through cell shredders (Macherey and Nagel, Oensingen, Switzerland) for 3 min at 11,000 g. After removing the shredders from the collecting tubes, the lysates were further centrifuged for 45 min at 20,000 g (4 °C). The resulting pellets were resuspended in membrane solubilization solution (25 μL per oocyte) containing (in mM): 50 Tris/HCl (pH 7.0 at RT), 10 N-ethylmaleimide, and 1% (v/v) Triton X100, supplemented with protease inhibitors as indicated above. The solubilization of membrane proteins was completed by incubating the homogenates for 45–60 min on an orbital shaker at 4 °C, followed by centrifugation for 12 min as before (= membrane-enriched fraction). Samples of these Triton-soluble fractions (total membrane fractions) were mixed with 4× Sample buffer (50 mM DTT final concentration) and heated for 5 min at 72 °C for SDS-PAGE separation and Western blot analysis. After adjusting the NaCl concentration to 150 mM (from a 5 M stock solution), the Biotinylated fractions were isolated from the membrane-enriched fractions and incubated on an orbital shaker for 5–6 h at 4 °C in the presence of 25 μL (bed volume) of streptavidin-agarose beads (Thermo Scientific #20353). Non-bound fractions were discarded, the beads were washed twice with RIPA buffer (Thermo Scientific #89901), supplemented with protease inhibitors, by incubating each time for 5 min on an orbital shaker at 4 °C. Beads were rinsed once with membrane solubilization solution supplemented with NaCl (150 mM) and protease inhibitors. The drained beads were finally resuspended in 50 μL of 2xSample buffer/DTT (50 mM final). Bound fractions were eluted by heating for 7 min at 72 °*C. Triton*-soluble fractions (1% of total) and neutravidin-bound fractions were resolved by SDS-PAGE, transferred to nitrocellulose membranes, and blocked in 2% (w/v) skimmed milk powder in 1xTBS. Nitrocellulose membranes were subsequently incubated overnight at 4 °C in the presence of antibodies (see Table 1) diluted in 1% (w/v) skimmed milk in 1xTBS. After three rounds of washing in 1xTBS, 0.05% Tween-20, membranes were incubated for 1 h at RT in the presence of HRP-conjugated anti-rabbit immunoglobulins (AffiniPure Fab Fragment Goat Anti-Rabbit, Jackson ImmunoResearch #111–007-003), diluted 1/12,000 in 1% (w/v) skimmed milk, in 1xTBS. After washing, the HRP signal was revealed using Western Bright Quantum detection reagent (Advansta, Menlo Park, CA, #K12042-D20) and detected using a Fusion Solo imaging system (Vilber Lourmat, Marne-la-Vallée, France). The exposure time was adjusted to avoid saturation of the recorded bands. Band intensities were measured from 16-bit, grayscale, uncompressed TIF images using ImageJ software (ImageJ, U.S. National Institutes of Health, Bethesda, Maryland, United States)^2^.

**Table 1:** Primary antibodies.

Antigen	Host	Source	Cat#	Dilution fold
ENaC α subunit	Rabbit	StressMarq	SPC-403S	1 k
ENaC β subunit	Rabbit	Jan Loffing (37)	N/A	10 k
ENaC γ subunit	Rabbit	StressMarq	SPC-405D	1 k
Na^+^/K^+^-ATPase, α subunit	Rabbit	K. Geering (38)	N/A	10 k

To correct for differences in protein recovery, the intensities of bands in membrane preparations and biotinylated fractions, corresponding to each of the ENaC subunits, were normalized to that of the endogenous Na^+^/K^+^-ATPase α subunit.

### 3.5 Statistical analysis

Statistical analysis was performed using GraphPad Prism, version 10 (RRID: SCR_002798). To compare two groups, the Student’s unpaired *t*-test or Mann Whitney test was used. For comparisons involving more than two groups, the Kruskal–Wallis test followed by Dunn’s multiple comparisons test was performed. The data are presented as mean ± standard error of the mean (SEM), showing the individual data points.

## 4 Results

### 4.1 The γENaC-G532S mutation results in ENaC current reduction

**Figure 1:**
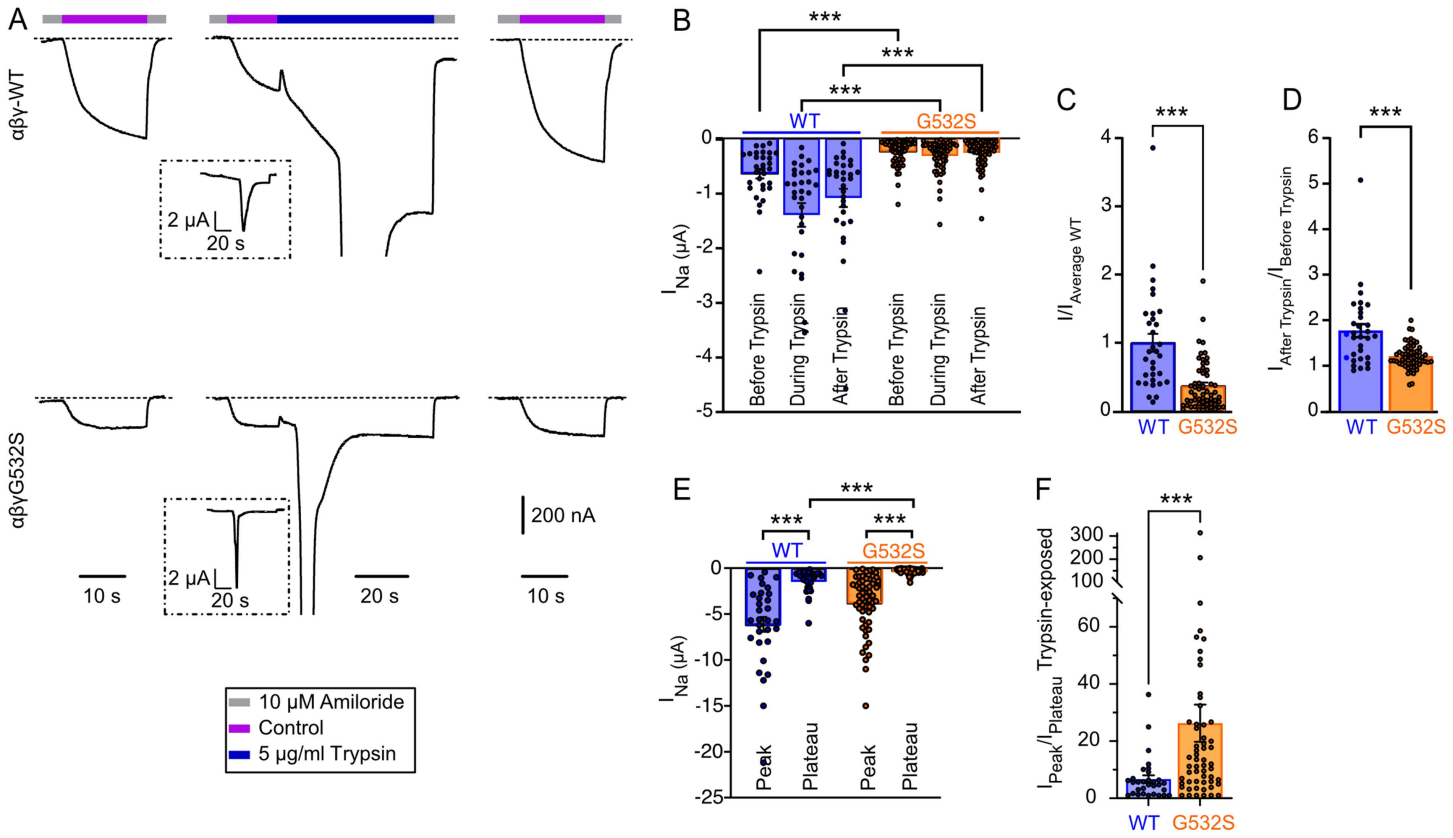
The γENaC-G532S mutation decreases the ENaC sodium current. **(A)** Current traces of αβγENaC-WT (top) and αβγENaC-G532S (bottom) are shown during exposure to the solutions indicated by the horizontal bars, at a holding potential of −80 mV. The left and right representative traces result from the protocol measuring amiloride-sensitive current; middle traces show currents measured during exposure to 5 μg/mL trypsin. The current amplitude scale is the same for the 6 traces, as indicated. **(B)** Amiloride-sensitive currents (I_Na_) measured in oocytes expressing αβγENaC-WT (*n* = 31) or αβγENaC-G532S (*n* = 57), measured before, during, and after treatment with trypsin. ***p < 0.001, Kruskal–Wallis’s one-way ANOVA test, followed by the Dunn’s multiple comparison test. **(C)** Normalized amiloride-sensitive currents. The current amplitudes recorded from a given batch of oocytes were normalized to the average ENaC-WT amplitude of that batch for this analysis. **(D)** Fold increase in I_Na_ measured after treatment with trypsin: in each oocyte, the amiloride-sensitive current amplitude after trypsin exposure was normalized to that measured before trypsin. **(E)** Amiloride-sensitive currents (I_Na_), peak and plateau, measured during treatment with trypsin. ***p < 0.001, Kruskal–Wallis’s one-way ANOVA test, followed by the Dunn’s multiple comparison test. **(F)** I_peak_/I_plateau_ of amiloride-sensitive current during trypsin treatment was calculated in each oocyte by normalizing the transient peak amplitude to the corresponding plateau current. **(C,D,F)** Mann–Whitney’s *U*-test was performed for statistical analysis. **(B–F)** Bars represent the mean ± SEM. Note that all data of this figure are from oocytes injected with RNA encoding the three subunits α, β and γ, αβγENaC-WT or αβγENaC-G532S.

The γENaC-Gly532 residue is highly conserved among ENaC/degenerin channels. Although the high-resolution 3D structures of ENaC do not resolve the parts containing this residue (9, 10), alignment to ASIC sequences indicate that this residue is located in the transmembrane α-helix 2 (TM2) which lines the channel pore. We examined here the functional consequences of the αβγG532S mutation. αβγWT or αβγG532S ENaC (i.e., αβγ ENaC containing the mutation G532S in the γ subunit) was expressed in *Xenopus laevis* oocytes, and channel function was measured with two-electrode voltage-clamp. Recordings were started in the presence of the ENaC inhibitor amiloride at a concentration of 10 μM, to establish the baseline. Subsequently, the oocytes were exposed for 20 s to an amiloride-free solution (control) and were then switched back to the amiloride-containing solution, to measure the amplitude of the ENaC current (left traces of Figure 1A). After repeating this protocol once, oocytes were exposed to the control solution for 20 s, followed by exposure to 5 μg/mL trypsin for 2 min (middle traces in Figure 1A). Trypsin, a serine protease, cleaves extracellular parts of α and γ subunits and removes inhibitory segments, thereby increasing ENaC activity (20–22). After washing out trypsin, the amiloride-sensitive current was measured again (traces on the right in Figure 1A). The amiloride-sensitive current amplitude of oocytes expressing the αβγG532S mutation was 37 ± 4% relative to that of oocytes expressing the αβγWT ENaC when measured before trypsin exposure (*p* < 0.0001; Figures 1B,C). In this analysis, the current amplitudes measured by individual αβγWT and αβγG532S ENaC oocytes were normalized with respect to the average amplitude of the αβγWT currents of the same oocyte batch.

A comparison of the current recorded before and after trypsin exposure provides an estimate of the open probability of ENaC channels under basal (unstimulated) conditions, under the assumption that after trypsin exposure, ENaC is fully active (8, 21, 23). αβγWT showed in many studies an open probability of ≤0.5 (24). Trypsin increased the ENaC αβγWT currents, as shown by the ratio of current after/before trypsin of 1.77 ± 0.14 (*n* = 31, Figures 1B,D). Assuming full activation after trypsin exposure, this indicates a basal open probability of ENaC αβγWT of 0.56. In contrast, the current ratio after/before trypsin was 1.2 ± 0.04 with αβγG532S (Figure 1D). The ratio is therefore 32% lower in the mutant (*p* ≤ 0.0001), suggesting an increased basal open probability in αβγG532S. Upon exposure to trypsin, both ENaC αβγWT and αβγG532S oocytes exhibited a transient current peak, followed by a stable plateau phase (Figure 1A), as observed in 78% of WT- and 90% of mutant channel-expressing oocytes (considering transient currents if I_peak_/I_plateau_ ≥ 1.5; Figure 1E). The transient peak current was 7 ± 1 times greater than the plateau current in αβγWT-expressing oocytes, and 26 ± 7 times greater in αβγG532S-expressing oocytes (Figure 1F). The trypsin-induced plateau, but not the transient current amplitude was significantly different between the WT and the mutant (Figure 1E). Taken together, the functional analysis shows a 63% current reduction in the mutant, which appears not to be caused by a lower open probability.

### 4.2 The γG532S mutation decreases ENaC expression

**Figure 2:**
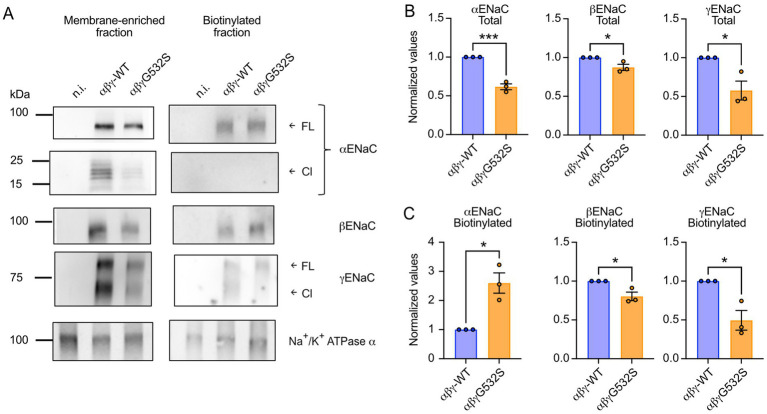
Biochemical analysis of WT and mutant ENaC. **(A)** Representative Western blot analysis of the total (=membrane-enriched) and the cell surface (biotinylated) fractions expression of each of the ENaC subunits, in oocytes injected with either αβγWT or αβγG532S cRNAs. Bands corresponding to the full-length (FL) or cleaved (Cl) forms of α- and γENaC are indicated. Western blots of the endogenous Na^+^/K^+^-ATPase α subunit were performed in parallel for normalization of protein recovery. **(B-C)** Densitometric quantification of Western blots from three independent experiments. The band intensities are normalized to the corresponding Na^+^/K^+^-ATPase band intensities to correct for differences in protein recovery. The values thus obtained for each experiment were normalized with that of the corresponding WT subunit. **(B)** Band intensities of each of the ENaC subunits in membrane-enriched fractions. **(C)** ENaC subunit expression from biotinylated fractions.

To assess whether the reduction in Na^+^ currents observed with the mutated form of the γENaC was a result of a decrease in total or cell surface expression of ENaC at the protein level, Western blot analysis was carried out from membrane-enriched fractions (= total expression) and from plasma membrane-resident channels isolated by cell surface biotinylation of intact oocytes, after injection with either αβγWT or αβγG532S cRNAs. Western blots of the membrane-enriched fractions showed that the oocytes expressing the mutated γ-ENaC displayed, when compared to the WT, a lower expression of not only this subunit, but also of the α and β subunits (Figures 2A,B). This decrease of expression at the protein level was confirmed on membrane-enriched fractions of oocytes injected only with cRNAs for either the wild type or the mutated γENaC subunit (Supplementary Figure S1), indicating that the effect of the mutation takes place before the formation of the three-subunit complex. Surprisingly, αENaC expression at the cell-surface was increased in the mutant αβγG532S (Figures 2A,C). Consistent with their total expression pattern and in contrast to αENaC, the expression at the cell surface of β- and γENaC was decreased in αβγG532S relative to αβγWT (Figures 2A,C). The biochemical analysis indicated the occurrence of cleavage of the α and γ subunits (Figure 2A). Although there may be an indication of lower abundance of the cleaved forms in oocytes expressing αβγG532S, the low expression of the mutant precluded a quantitative analysis of subunit cleavage.

## 5 Discussion

We describe here the case of an infant born out of a consanguineous marriage, who exhibited features indicative of mild PHA-1 due to a novel mutation in the SCNN1G gene. The mild phenotype exhibited by the infant was reminiscent of PHA-1A, with the symptoms easily managed with low-dose salt supplementation. PHA-1A is a renal limited, autosomal dominant condition, known to present with a mild phenotype, whereas PHA-1B is an autosomal recessive, severe multisystem disorder. A mild phenotype of PHA-1B has so far only been reported in the case of a missense mutation in the SCNN1A gene (18). This is the first time that a missense mutation in SCNN1G gene has been identified in a child presenting with a mild form of PHA-1B. Our *in vitro* analysis provides evidence for the pathogenic role of the SCNN1G mutation p. Gly532Ser. This analysis was carried out in *Xenopus* oocytes, a cell type that is different from epithelial cells where ENaC is typically expressed. This expression system is suitable for the study of ENaC, as shown by many studies (7, 13). Although some regulatory mechanisms are different between the cell system used here and epithelial cells, we observed in direct comparison a clear difference in the expression and the basic channel function between the wild type and mutant ENaC form, supporting a significant functional effect of the γG532S mutation on ENaC expression and function.

The functional ENaC contains the three subunits α, β, and γ. The current amplitude is drastically decreased if one subunit is missing or non-functional (16, 25). Currently known PHA-1B-associated mutations occur in all three ENaC subunits (5). Most γENaC PHA-1B mutations reported so far change the gene structure by inducing truncations or affecting the splicing (17, 26–28). The previously reported PHA-1B-inducing missense mutation ENaC-αβγA63P (6) changed a residue in the central part of the first transmembrane segment (TM1). In ENaC/degenerin channels, the pore is lined by the TM2, while the TM1 is positioned peripherally (10, 29, 30). The A63P mutation completely changed the side chain properties at this position, likely inducing a strong reduction of ENaC channel function. The ENaC-αβγG532S mutation reported here induces smaller changes in side chain properties; however, the mutation occurs in a highly conserved, pore-lining residue, where small changes in side chain properties are expected to significantly change channel function (31). Substitution at the same position by Cys, thus the ENaC-αβγG532C mutation, had previously been shown not to affect the Li^+^/Na^+^ and K^+^/Na^+^ ion selectivity, but to decrease the amiloride-sensitive current amplitude by 14% (32). Here we show that the αβγENaC-G532S mutation resulted in a reduction of the ENaC-mediated current amplitude of 63 ± 5%.

This current reduction is at least in part caused by an effect on the ENaC expression level. We show that the γG532S mutation decreases the protein expression level of γENaC. This reduction is accompanied by a concomitant decrease in the total expression of both the α and β subunits (Figure 2). Since the expression of γG532S is also lower than that of γWT in the absence of the α and β subunits, we hypothesize that the residue substitution at position 532 might be detected by the ER-associated degradation (ERAD) pathway. Indeed, proteins with folding lesions within the transmembrane domains, termed ERAD-M substrates, have been shown to be ubiquitylated by Hrd1 (33). Since the assembly of the α, β, and γENaC into a trimeric complex stabilizes the three subunits (34, 35), it is possible that a reduced half-life of γG532S has repercussions on the stability of the two other subunits, hence providing a possible explanation for the lower expression levels of α and βENaC in oocytes co-injected with of γG532S. Furthermore, this effect could be enhanced if the mutated γ subunit has a reduced capacity to associate with the α and β subunits to form a canonical complex. It is surprising that at the cell surface, αENaC expression is higher in mutant- than in WT-expressing cells. However, due to the lower abundance of β and γ subunits at the cell surface, there are less trimeric αβγENaC channels present in the plasma membrane of αβγG532S-expressing cells. This lower abundance of αβγ trimers at the plasma membrane causes most likely the observed lower current amplitudes in αβγG532S- as compared to αβγWT-expressing cells.

PHA1-1B typically manifests in the first days of life as a severe disease requiring supplementation of high quantities of Na^+^-containing salts and K^+^-absorbing resins for many years or even lifelong (28, 36). The infant reported here was brought to the clinic only at 4 months of age. While there was definite hyponatremia and increased aldosterone and renin levels, and it presented with failure to gain weight, the hyperkalemia was mild, and the infant was not dehydrated, nor did it present a skin or pulmonary phenotype. The infant has been on just 10 meq/kg of daily salt supplementation, and on follow-up over 1 year, continues to grow well and is maintaining normal biochemical parameters on this therapy.

## 6 Conclusion

This study highlights a mild course of PHA-1B associated with a missense mutation in the ENaC γ subunit (SCNN1G). The biochemical and functional *in vitro* study shows that the γG532S mutation decreases but does not disrupt ENaC expression and function.

## Data Availability

The original contributions presented in the study are included in the article/Supplementary material, further inquiries can be directed to the corresponding author.

## Footnotes

^1^ https://omim.org/

^2^ http://imagej.nih.gov/ij/
