# Supplementary material for: Case Report: Functional investigation of the γENaC G532S mutation presenting as mild PHA-1B3
Source: Front Med (Lausanne). 2025 Sep 3;12:1605057. doi: 10.3389/fmed.2025.1605057 (PMC12440858; doi:10.3389/fmed.2025.1605057)
Supplement: Supplementary file 1 [file Data_Sheet_1.pdf]

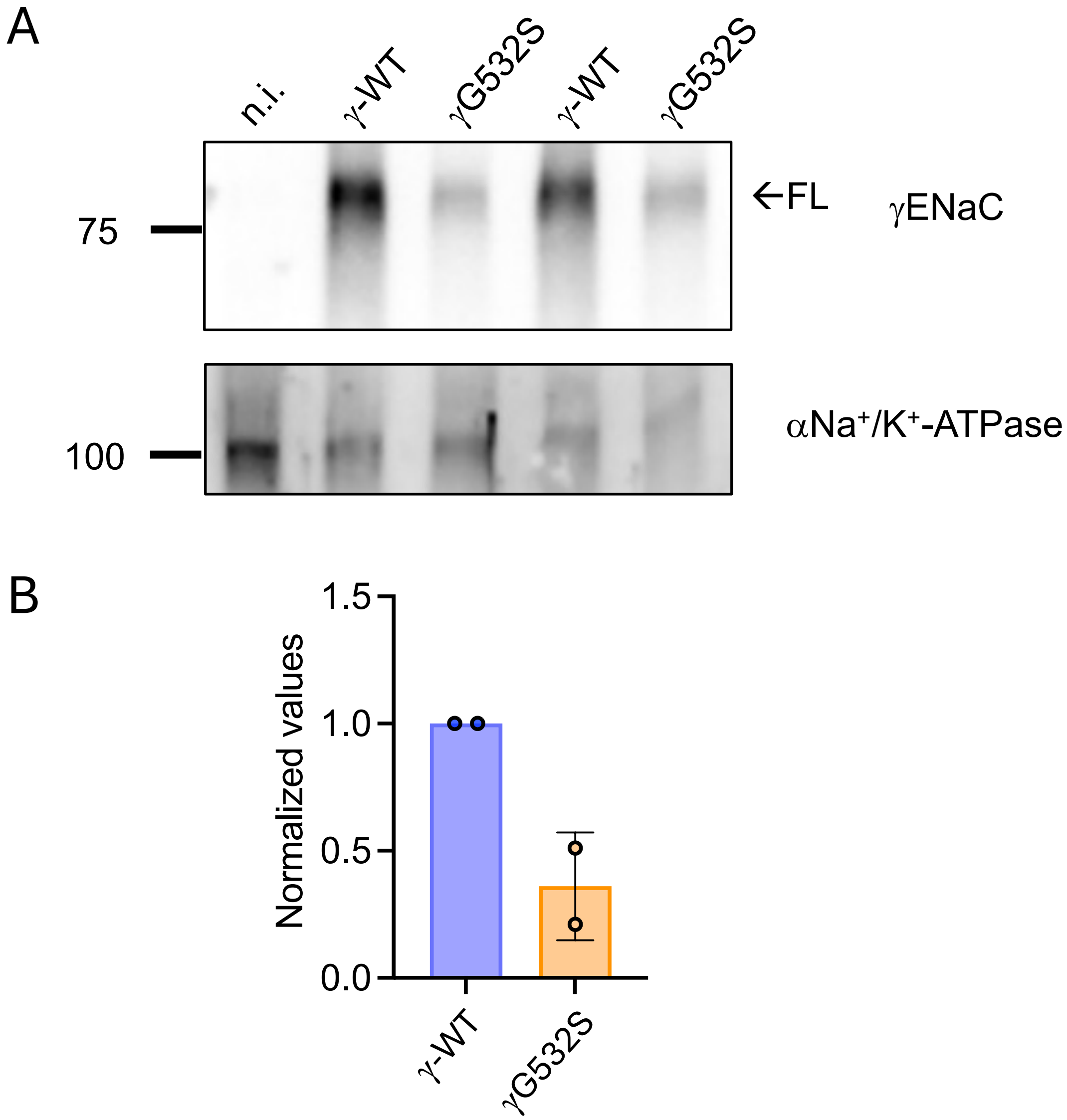

Supplementary Figure S1. The G532S mutation decreases expression of γENaC if expressed without α and β subunits. A. Representative western blot analysis of the total expression, in oocytes injected with either γWT or γG532S cRNAs. Bands corresponding to the full-length (FL) form of γENaC are indicated. Western blots of the endogenous Na^+/K^+-ATPase α subunit were performed in parallel for normalization of protein recovery. B. Densitometric quantification of western blots from two independent experiments. The band intensities of each of the ENaC subunits in membrane-enriched fractions, were normalized with the corresponding Na^+/K^+-ATPase band intensities to correct for differences in protein recovery. The values thus obtained for each experiment were normalized with that of the WT subunit.
