# Supplementary material for: Case Report: Functional investigation of the γENaC G532S mutation presenting as mild PHA-1B3
Source: Front Med (Lausanne). 2025 Sep 3;12:1605057. doi: 10.3389/fmed.2025.1605057 (PMC12440858; doi:10.3389/fmed.2025.1605057)

Note: certain experiments have been carried out in parallel with oocytes in which the non-modified form of alpha ENaC was replaced by an HA-tagged form of this protein, for detection with anti-HA-tag antibodies. Westerns made using an anti-human alpha ENaC subunit showed that the expression levels of the tagged form was around five times lower than that of the non-tagged form. Because the possibility that the alpha-HA is limiting, these data haven't been included in the manuscript.

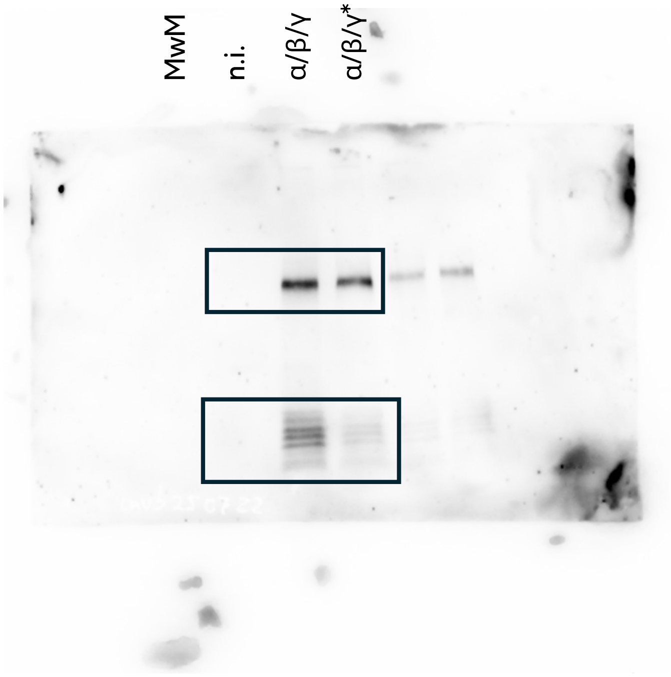

Alpha ENaC biotinylated fractions

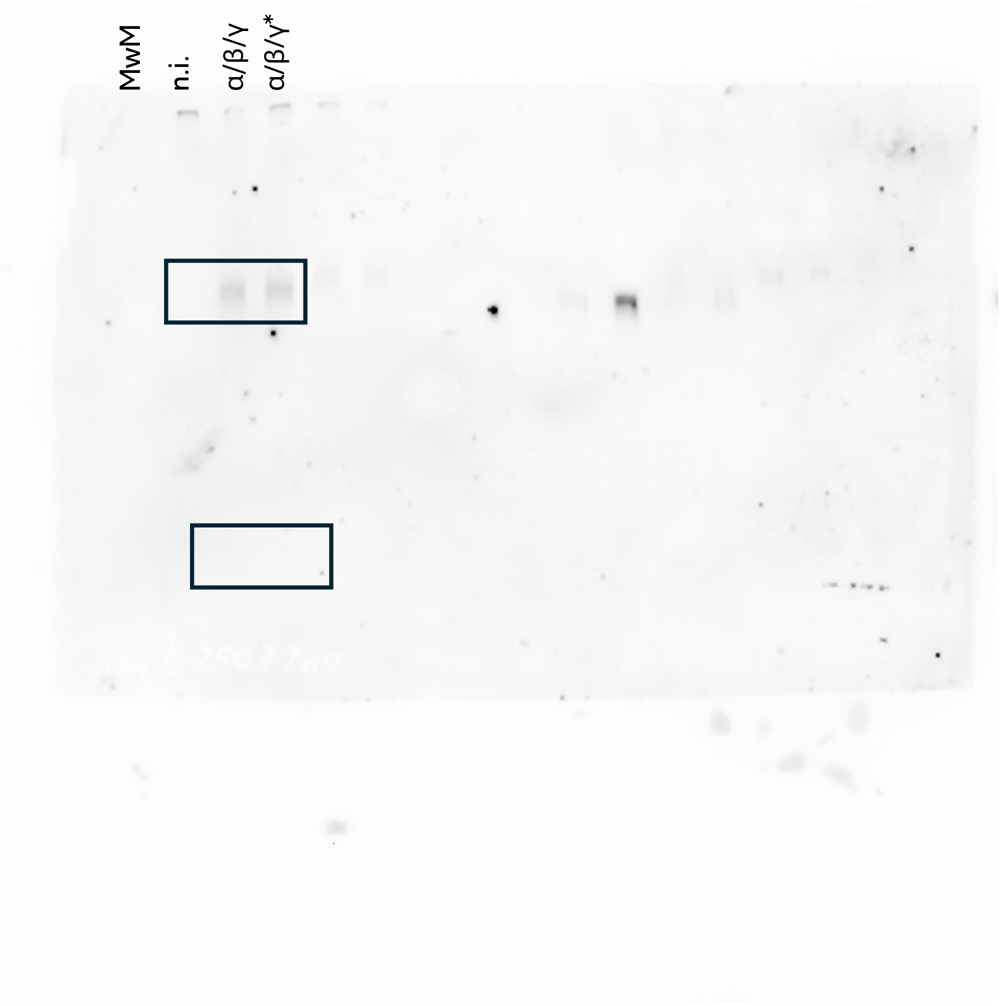

Beta ENaC membrane-enriched, Triton-soluble fractions

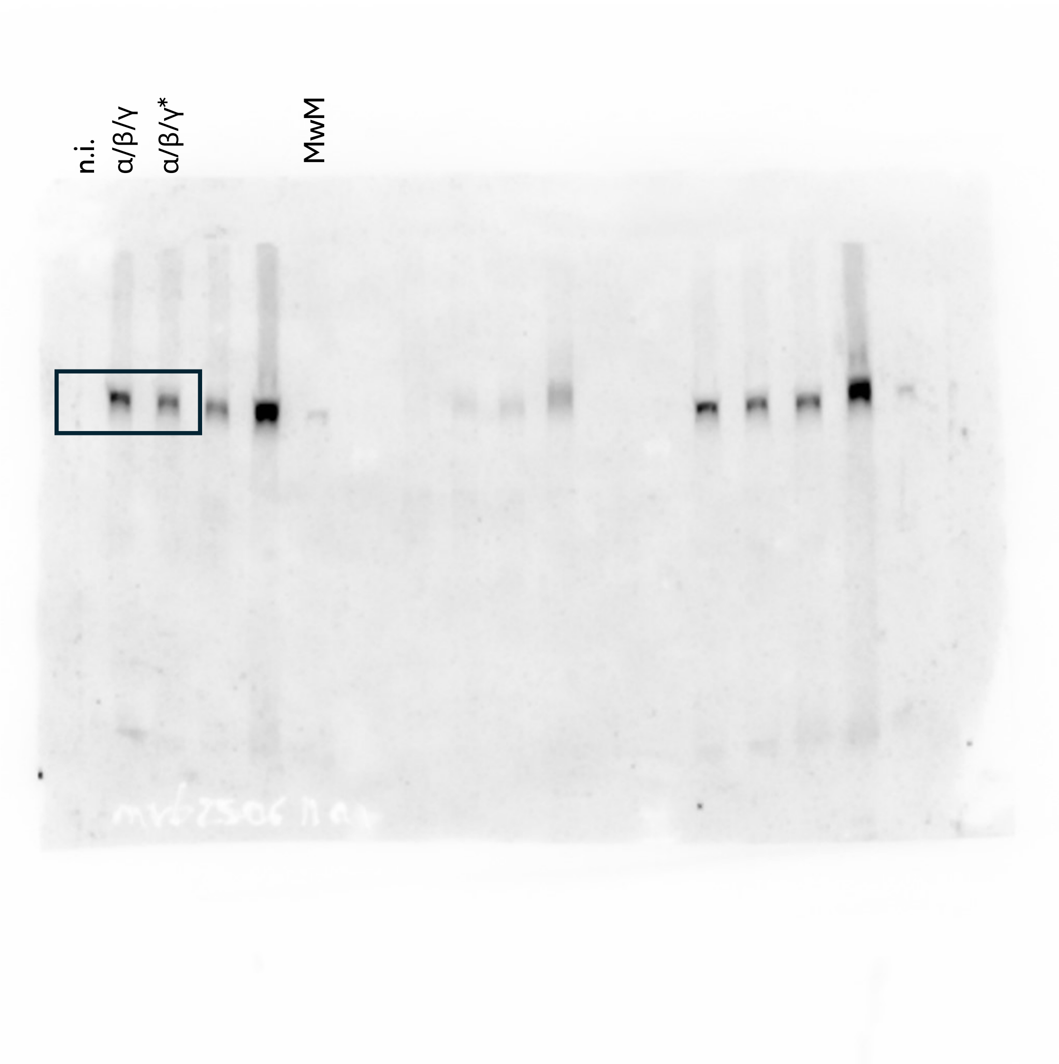

Beta ENaC biotinylated fractions

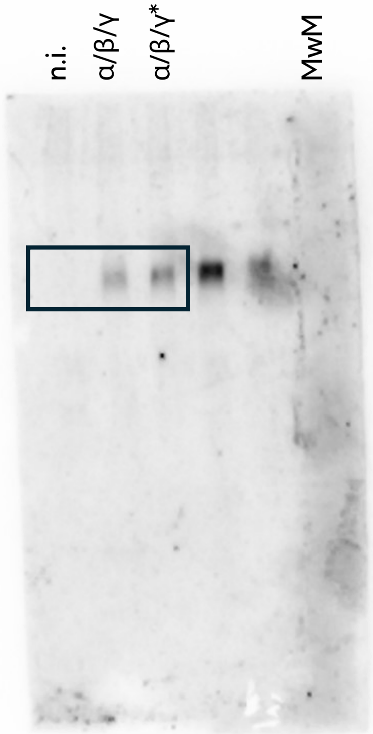

Gamma ENaC membrane-enriched, Triton-soluble fractions

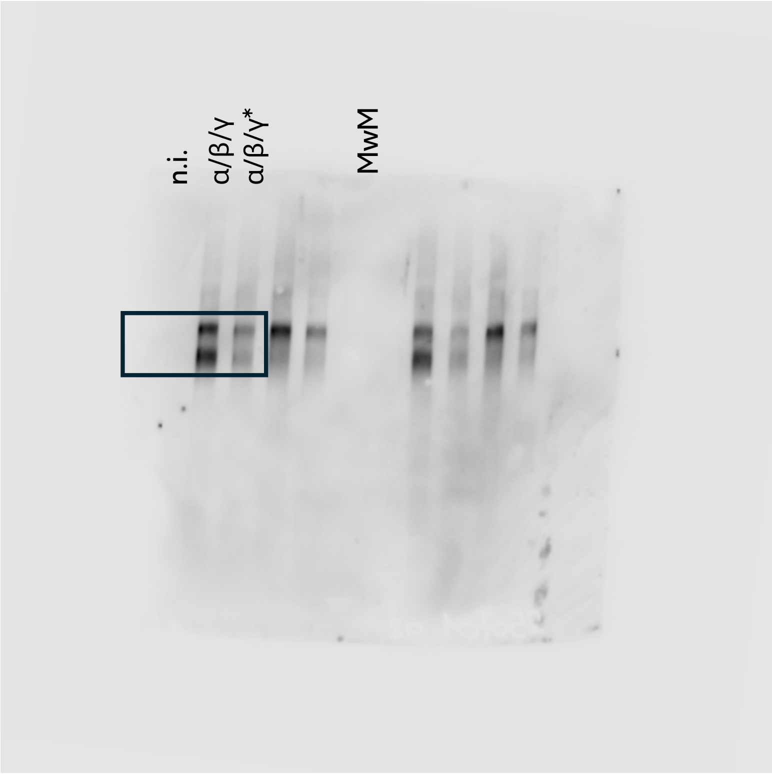

Gamma ENaC, biotinylated fractions

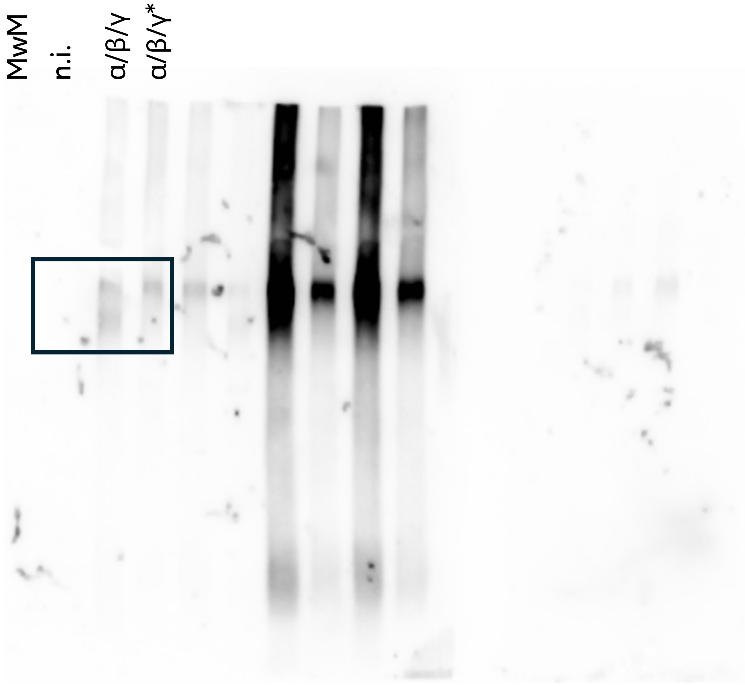

Na+/K+-ATPase alpha subunit. membrane-enriched, Triton-soluble fractions

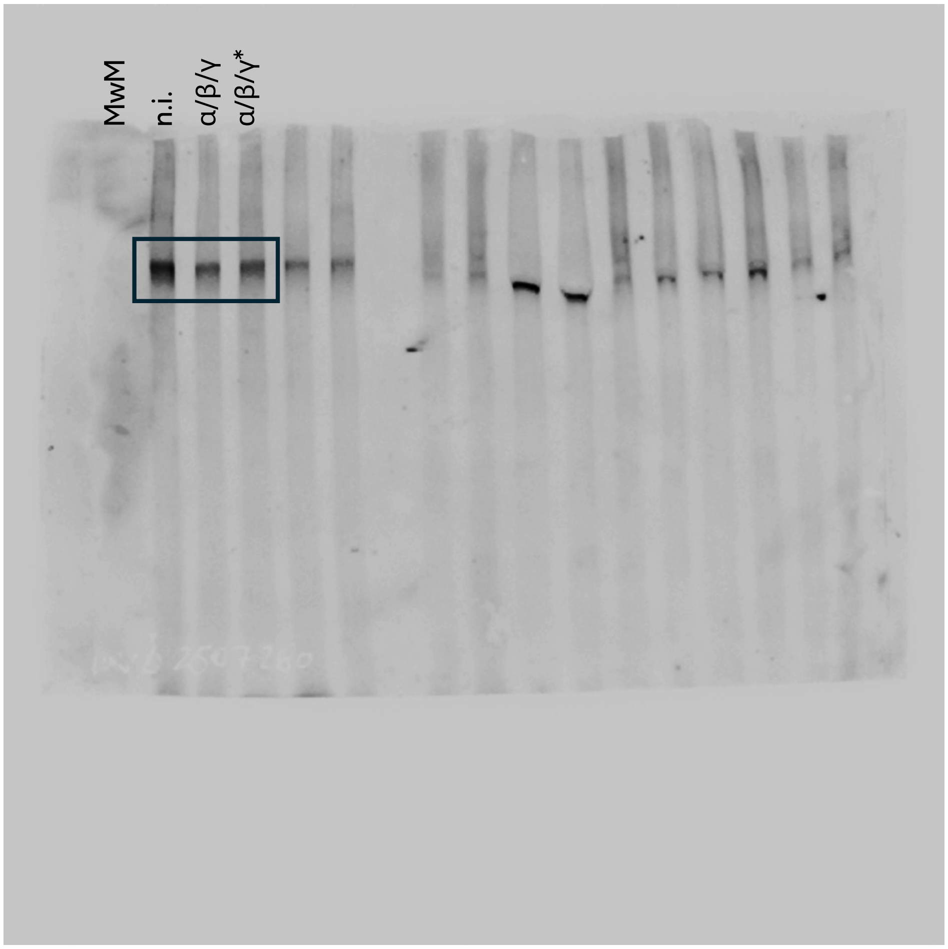

Na+/K+-ATPase alpha subunit, biotinylated fractions

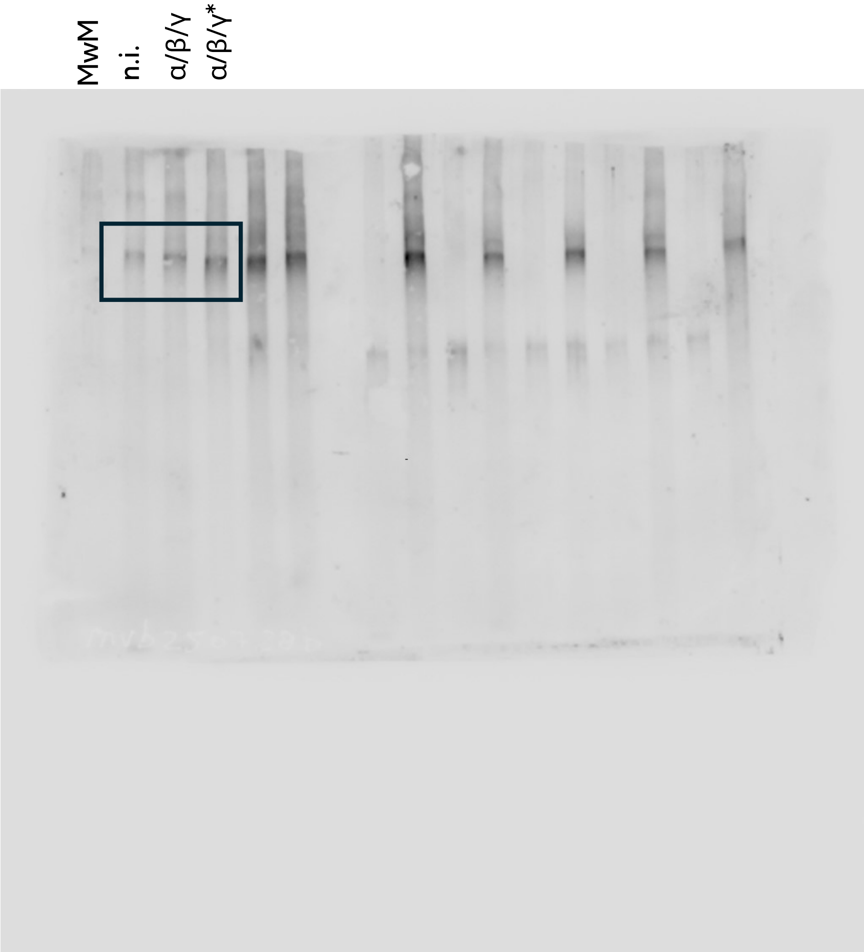

Original images Supplementary figure

Gamma ENaC membrane-enriched, Triton-soluble fractions

Anti gamma ENaC

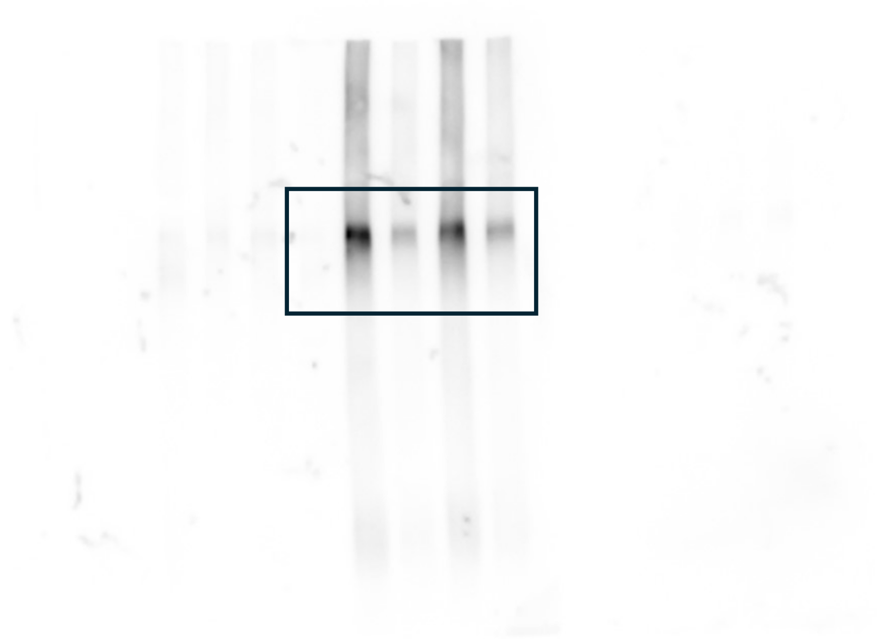

Original images Supplementary figure

Gamma ENaC membrane-enriched, Triton-soluble fractions

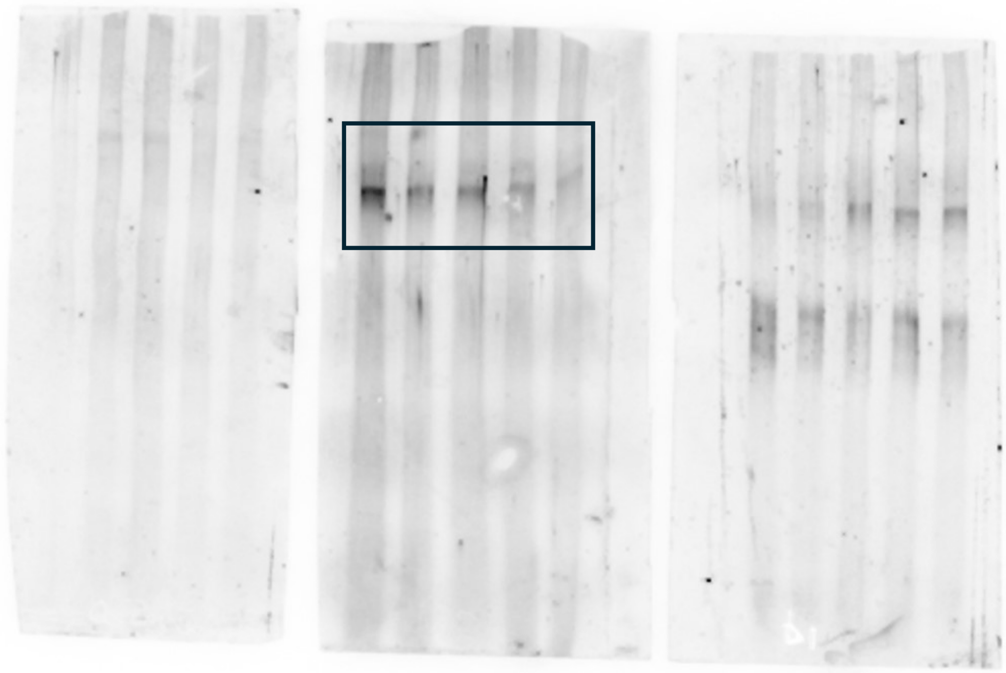

Supplement: Supplementary file 2 [file Data_Sheet_2.pdf]
